# Type 2 diabetes and in-hospital sudden cardiac arrest in ST-elevation myocardial infarction in the US

**DOI:** 10.3389/fcvm.2023.1175731

**Published:** 2023-07-03

**Authors:** Omar Mhaimeed, Krishnadev Pillai, Soha Dargham, Jassim Al Suwaidi, Hani Jneid, Charbel Abi Khalil

**Affiliations:** ^1^ Johns Hopkins Hospital, Osler Medical Residency, Johns Hopkins University, Baltimore, MD, United States; ^2^ Department of Medicine, Weill Cornell Medicine-Qatar, Doha, Qatar; ^3^ Biostatistics Core, Weill Cornell Medicine-Qatar, Doha, Qatar; ^4^ Heart Hospital, Hamad Medical Corporation, Doha, Qatar; ^5^ Department of Internal Medicine, University of Texas Medical Branch (UTMB), Galveston, TX, United States; ^6^ Joan and Sanford I. Weill Department of Medicine, Weill Cornell Medicine, New York, NY, United States

**Keywords:** ST elevation myocardial infarction, sudden cardiac arrest, diabetes, mortality, cardiovascular disease

## Abstract

**Aims:**

We aimed to assess the impact of diabetes on sudden cardiac arrest (SCA) in US patients hospitalized for ST-elevation myocardial infarction (STEMI).

**Methods:**

We used the National Inpatient Sample (2005–2017) data to identify adult patients with STEMI. The primary outcome was in-hospital SCA. Secondary outcomes included in-hospital mortality, ventricular tachycardia (VT), ventricular fibrillation (VF), cardiogenic shock (CS), acute renal failure (ARF), and the revascularization strategy in SCA patients.

**Results:**

SCA significantly increased from 4% in 2005 to 7.6% in 2018 in diabetes patients and from 3% in 2005 to 4.6% in 2018 in non-diabetes ones (*p* < 0.001 for both). Further, diabetes was associated with an increased risk of SCA [aOR = 1.432 (1.336–1.707)]. In SCA patients with diabetes, the mean age (SD) decreased from 68 (13) to 66 (11) years old, and mortality decreased from 65.7% to 49.3% during the observation period (*p* < 0.001). Compared to non-diabetes patients, those with T2DM had a higher adjusted risk of mortality, ARF, and CS [aOR = 1.72 (1.62–1.83), 1.52 (1.43–1.63), 1.25 (1.17–1.33); respectively] but not VF or VT. Those patients were more likely to undergo revascularization with CABG [aOR = 1.197 (1.065–1.345)] but less likely to undergo PCI [aOR = 0.708 (0.664–0.754)].

**Conclusion:**

Diabetes is associated with an increased risk of sudden cardiac arrest in ST-elevation myocardial infarction. It is also associated with a higher mortality risk in SCA patients. However, the recent temporal mortality trend in SCA patients shows a steady decline, irrespective of diabetes.

## 1. Introduction

Sudden cardiac arrest (SCA) is the most common cause of death in the United States and worldwide. In the general population, SCA accounts for approximately 10% of total mortality and 40% of mortality from coronary artery disease (CAD) (1, 2). Mortality rates from SCA increase with age and are reported to be higher in males compared to females and higher in African-Americans compared to Caucasians (3, 4).

SCA is often the consequence of lethal arrhythmias, most commonly ventricular fibrillation (5). Inciting events could be due to an acute atherosclerotic plaque rupture, electrolyte imbalance, and increased sympathetic activity in patients with underlying structural and coronary heart disease (6).

During the past two decades, type 2 diabetes mellitus (T2DM) reached epidemic proportions worldwide. Diabetes is an established risk factor for cardiovascular disease; it is also associated with higher mortality and morbidity in patients with established atherosclerotic cardiovascular disease (ASCVD) (7). However, the landscape of diabetes has changed over the past decade. Despite the rise in the incidence of diabetes and its related complication, mortality in the general population is overall decreasing (8); which is also the case in clinical conditions such as heart failure, stroke, valvular heart disease, and hypertrophic cardiomyopathy (9–12).

In this study, we report a temporal increase in the number of in-hospital SCA in STEMI patients. However, in-hospital mortality in those patients is on a descending slope. Nonetheless, diabetes is still associated with a higher risk of SCA and mortality.

## 2. Methods

### 2.1. Data source

The National Inpatient Sample (NIS) database was used to obtain patient data from 2005 to 2017. The NIS is an administrative and de-identified database designed by the Healthcare Cost and Utilization Project (HCUP) to produce US regional and national estimates of inpatient utilization, access, charges, quality, and outcomes. It accounts for 20% of all US community hospitals. Each entry in the database entails demographic details such as age, sex, race, etiology of admissions, outcomes, and procedures while using safeguards to protect the privacy of patients, physicians, and hospitals (13). Each admission consists of unique identifiers in the database, preventing repeat admissions for the same patient from being distinguished. The study did not require institutional review board approval but an exempt determination (number 18-00017).

### 2.2. Study population

The ICD-9-CM and ICD-10-CM (International Classification of Diseases, Ninth and Tenth Revisions, Clinical Modification) codes were used to identify admissions of patients from the years 2005–2017 with a primary diagnosis of STEMI who developed in-hospital SCA. Further, patients were categorized according to the presence of diabetes. Patients less than 18 years of age and those with incomplete or missing data were excluded.

### 2.3. Outcomes

The primary outcome of this study was in-hospital SCA. Secondary outcomes include in-hospital mortality, ventricular fibrillation (VF), ventricular tachycardia (VT), acute renal failure (ARF), and cardiogenic shock in SCA patients. We further assessed interventional procedures: percutaneous coronary intervention (PCI) and coronary artery bypass graft (CABG). Diagnoses were based on ICD-9 and ICD-10 codes.

### 2.4. Analysis plan and statistics

Data were weighted as mandated by the HCUP to generate national estimates of admissions each year (14). We first analyzed the characteristics and outcomes of all STEMI patients who develop SCA. We then stratified them into two groups according to the presence of T2DM. Further, we merged both groups for intercomparison during the observation period. Finally, we assessed the predictors of mortality in patients with diabetes. Data are presented as mean (SD), median (IQR), number (%), or OR (95% CI). A simple linear regression was used to identify trends. A Chi-squared test, student *T*-test, or ANOVA was used to compare groups, as appropriate. Binary logistic regression was used to calculate the unadjusted odds ratios of outcomes, which were further adjusted for all baseline characteristics that were different among both groups. Multivariate logistic regression was performed to determine predictors of mortality. It included age, gender, race, hypertension, obesity, smoking, dyslipidemia, chronic kidney disease (CKD), and CAD. The significance level was set at 5%; all analyses were done using SPSS version 26 (IBM SPSS Statistics, IBM, Armonk, New York).

## 3. Results

### 3.1. Study group

**Figure 1:**
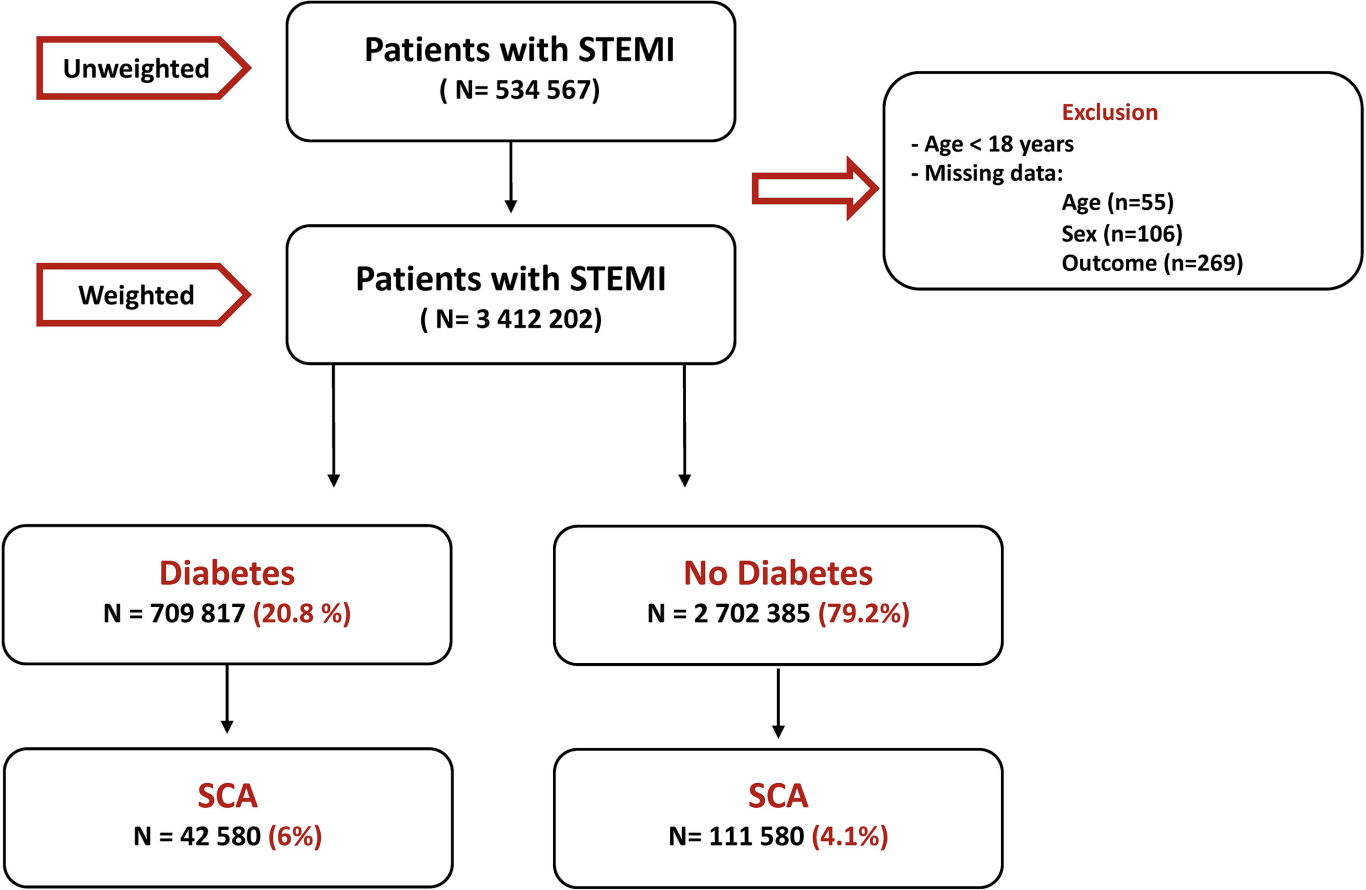
Flow chart of the study.

A total of 543,094 patients were hospitalized with the primary diagnosis of STEMI between 2005 and 2017. After excluding patients below 18 years old and those with missing or incomplete records, the dataset consisted of 534,567 patients (Figure 1). Weighted, 3,412,202 patients with STEMI were included in the initial analysis. Among those, 20.8% had diabetes.

### 3.2. Temporal trend in characteristics and outcomes of patients with STEMI who develop SCA

**Figure 2:**
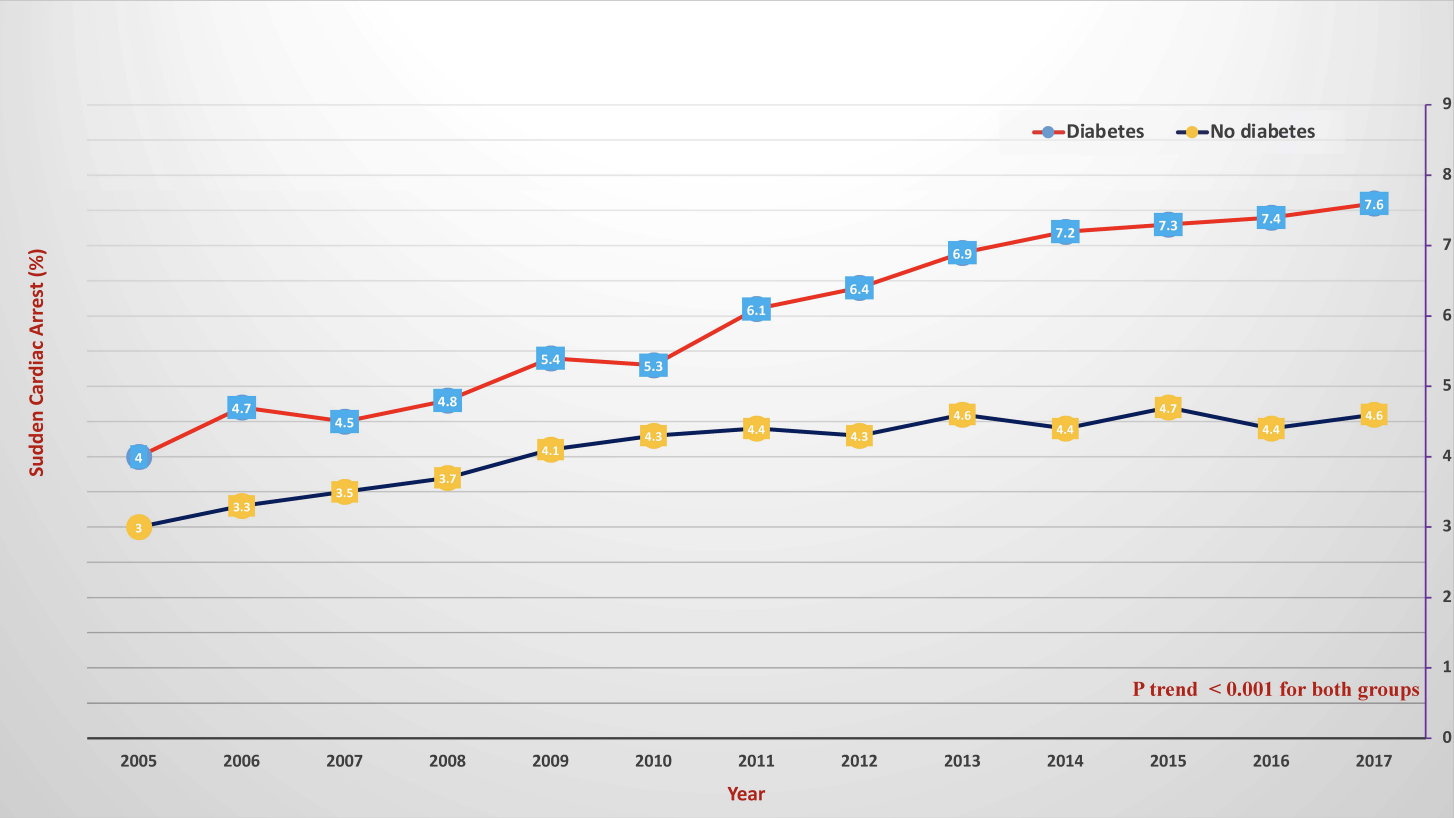
Temporal changes of in-hospital sudden cardiac arrest in patients with diabetes (red color) and without diabetes (blue color). The *X*-axis represents the percentage of sudden cardiac arrest. The *Y*-axis represents the year.

**Figure 3:**
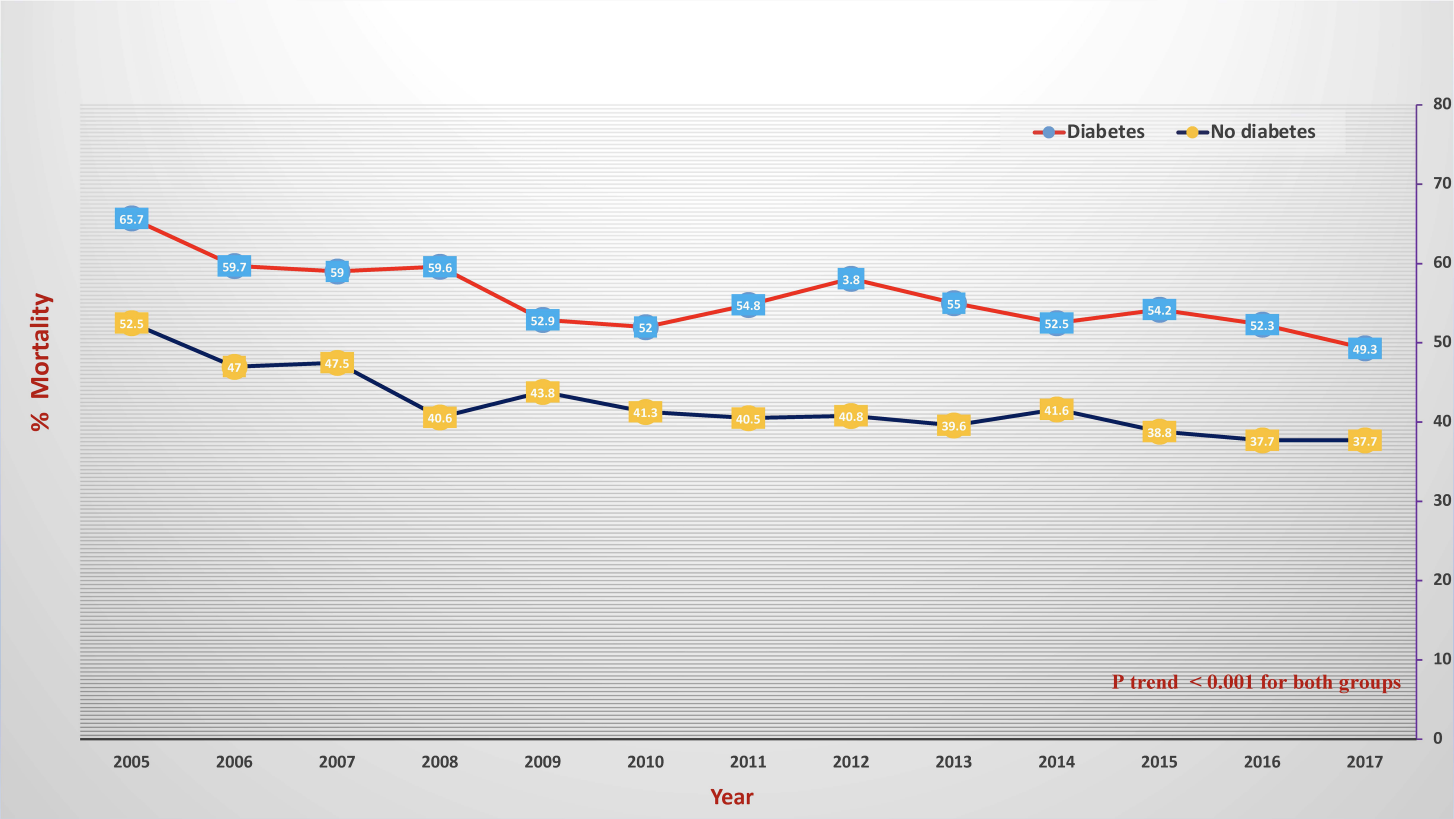
Temporal change of in-hospital mortality in patients with sudden cardiac arrest and diabetes (red color) and without diabetes (blue). The *X*-axis represents the percentage of sudden mortality. The *Y*-axis represents the year.

During the observation period, SCA was recorded in 6% of diabetes patients and 4.1% of non-diabetes patients (Figure 1); hence, diabetes significantly increased the risk of SCA [OR = 1.451, 95% CI (1.439–1.477)] even after correcting for confounding factors [adjusted OR = 1.432 (1.336–1.707)]. SCA significantly increased from 4% in 2005 to 7.6% in 2018 in diabetes patients and from 3% in 2005 to 4.6% in 2018 in non-diabetes ones (*p* < 0.001 for both) (Figure 2). Over the observation period, the mean age (SD) of patients with STEMI, SCA, and T2DM decreased from 68 (13) to 66 (11) (Table 1, *p* = 0.003). The proportion of patients in the age categories of <55, 55–64, and 65–74 increased over time, while that of patients in the age categories of 75–84 and >85 decreased (*p* < 0.001 for all). The patients were predominantly males throughout the years, and the proportion of male patients increased from 59.0% to 69.1% (*p* trend < 0.001). The proportion of Caucasian patients decreased, whereas that of African-Americans rose from 8.0% to 12.1% (*p* trend <0.001). The prevalence of comorbidities such as hypertension, obesity, smoking, dyslipidemia, CAD, and CKD also increased over time (*p* < 0.001). Mortality decreased from 65.7% to 49.3%, which was also observed in-non diabetes patients (Figure 3, *p* < 0.001 for both). There was a temporal increase in the proportions of patients who developed VT, VF, acute renal failure, and cardiogenic shock (*p* < 0.001 for all). As expected, a temporal increase in the prevalence of PCI was observed, parallel to a decrease in the rate of CABG (*p* < 0.001). The same baseline characteristics and outcomes trend was also observed in non-diabetes patients (Table 2).

**Table 1:** Trends in demographics, comorbidities, outcomes, and interventions in patients with STEMI, SCA, and diabetes.

Year	2005	2006	2007	2008	2009	2010	2011	2012	2013	2014	2015	2016	2017	*p* trend
Age														
Mean (SD)	68 (13)	68 (13)	67 (13)	67 (13)	66 (13)	67 (12)	67 (12)	67 (12)	66 (12)	66 (12)	66 (12)	66 (12)	66 (11)	0.003
Age: <55	381 (15.8%)	457 (16.1%)	489 (18.0%)	500 (16.8%)	625 (20.0%)	472 (16.3%)	518 (15.9%)	520 (15.3%)	570 (15.6%)	805 (18.8%)	760 (17.3%)	620 (17.4%)	540 (17.6%)	<0.001
Age: 55–64	532 (22.1%)	680 (23.9%)	584 (21.5%)	832 (28.0%)	780 (24.9%)	804 (27.8%)	934 (28.7%)	975 (28.7%)	1,095 (30.0%)	1,145 (26.8%)	1,310 (29.8%)	1,080 (30.3%)	835 (27.2%)	<0.001
Age: 65–74	606 (25.2%)	725 (25.5%)	789 (29.0%)	725 (24.4%)	829 (26.5%)	790 (27.3%)	819 (25.1%)	970 (28.5%)	1,080 (29.6%)	1,160 (27.1%)	1,255 (28.6%)	965 (27.1%)	975 (31.7%)	<0.001
Age: 75–84	671 (27.9%)	723 (25.4%)	631 (23.2%)	659 (22.2%)	615 (19.7%)	632 (21.9%)	719 (22.1%)	675 (19.9%)	650 (17.8%)	835 (19.5%)	780 (17.8%)	635 (17.8%)	570 (18.5%)	<0.001
Age: >85	217 (9.0%)	255 (9.0%)	230 (8.4%)	253 (8.5%)	279 (8.9%)	194 (6.7%)	270 (8.3%)	260 (7.6%)	255 (7.0%)	335 (7.8%)	285 (6.5%)	265 (7.4%)	155 (5.0%)	<0.001
Gender														
Male	1,420 (59.0%)	1,698 (59.8%)	1,659 (61.0%)	1,748 (58.9%)	1,939 (62.0%)	1,849 (64.0%)	2,057 (63.1%)	2,100 (61.8%)	2,350 (64.4%)	2,680 (62.6%)	2,975 (67.8%)	2,385 (66.9%)	2,125 (69.1%)	<0.001
Race														
Caucasians	1,356 (71.0%)	1,528 (70.7%)	1,428 (69.6%)	1,716 (71.8%)	2,016 (73.9%)	1,604 (65.6%)	2,014 (69.2%)	2,110 (66.9%)	2,425 (69.9%)	2,800 (69.7%)	2,760 (67.3%)	2,310 (67.5%)	2,010 (67.6%)	<0.001
African Americans	153 (8.0%)	205 (9.5%)	256 (12.5%)	236 (9.9%)	244 (8.9%)	262 (10.7%)	297 (10.2%)	370 (11.7%)	325 (9.4%)	415 (10.3%)	530 (12.9%)	440 (12.9%)	360 (12.1%)	<0.001
Hispanics	252 (13.2%)	273 (12.6%)	218 (10.6%)	247 (10.3%)	270 (9.9%)	327 (13.4%)	360 (12.4%)	420 (13.3%)	405 (11.7%)	485 (12.1%)	480 (11.7%)	415 (12.1%)	385 (12.9%)	<0.001
Asians	75 (3.9%)	73 (3.4%)	54 (2.6%)	62 (2.6%)	73 (2.7%)	96 (3.9%)	90 (3.1%)	90 (2.9%)	110 (3.2%)	105 (2.6%)	140 (3.4%)	100 (2.9%)	100 (3.4%)	<0.001
Comorbidities														
Hypertension	1,400 (58.2%)	1,649 (58.0%)	1,644 (60.4%)	1,955 (65.8%)	2,175 (69.5%)	2,036 (70.4%)	2,362 (72.5%)	2,600 (76.5%)	2,815 (77.1%)	3,160 (73.8%)	3,390 (77.2%)	2,820 (79.1%)	2,575 (83.7%)	<0.001
Obesity	279 (11.6%)	331 (11.7%)	306 (11.2%)	514 (17.3%)	537 (17.2%)	470 (16.2%)	633 (19.4%)	750 (22.1%)	770 (21.1%)	920 (21.5%)	1,025 (23.3%)	860 (24.1%)	730 (23.7%)	<0.001
Smoking	294 (12.2%)	445 (15.7%)	443 (16.3%)	520 (17.5%)	818 (26.1%)	863 (29.8%)	858 (26.3%)	1,030 (30.3%)	1,170 (32.1%)	1,620 (37.9%)	1,360 (31.0%)	600 (16.8%)	555 (18.0%)	<0.001
Dyslipidemia	659 (27.4%)	769 (27.1%)	904 (33.2%)	1,069 (36.0%)	1,394 (44.6%)	1,457 (50.4%)	1,673 (51.3%)	1,785 (52.5%)	1,960 (53.7%)	2,380 (55.6%)	2,475 (56.4%)	1,935 (54.3%)	1,685 (54.8%)	<0.001
CAD	1,348 (56.0%)	1,641 (57.7%)	1,585 (58.2%)	1,957 (65.9%)	2,271 (72.6%)	2,277 (78.7%)	2,468 (75.7%)	2,615 (76.9%)	2,825 (77.4%)	3,370 (78.7%)	3,465 (79.0%)	3,510 (80.1%)	3,545 (80.5%)	<0.001
CKD	356 (14.8%)	602 (21.2%)	570 (20.9%)	637 (21.5%)	765 (24.5%)	660 (22.8%)	807 (24.8%)	940 (27.6%)	970 (26.6%)	975 (22.8%)	1,155 (26.3%)	1,045 (29.3%)	855 (27.8%)	<0.001
Events														
VT	550 (22.8%)	587 (20.7%)	735 (27.0%)	670 (22.6%)	694 (22.2%)	807 (27.9%)	896 (27.5%)	875 (25.7%)	890 (24.4%)	1,190 (27.8%)	1,400 (31.9%)	1,244 (34.9%)	1,328 (43.2%)	<0.001
VF	753 (31.3%)	904 (31.8%)	886 (32.6%)	1,084 (36.5%)	1,035 (33.1%)	1,061 (36.7%)	1,182 (36.2%)	1,205 (35.4%)	1,420 (38.9%)	1,690 (39.5%)	1,874 (42.7%)	1,426 (40.0%)	1,306 (42.5%)	<0.001
Acute renal failure	468 (19.4%)	615 (21.6%)	762 (28.0%)	756 (25.5%)	1,109 (35.4%)	974 (33.7%)	1,296 (39.7%)	1,470 (43.2%)	1,500 (41.1%)	1,780 (41.6%)	2,010 (45.8%)	1,590 (44.6%)	1,495 (48.6%)	<0.001
Cardiogenic shock	733 (30.4%)	893 (31.4%)	900 (33.1%)	1,038 (35.0%)	1,189 (38.0%)	1,230 (42.5%)	1,578 (48.4%)	1,595 (46.9%)	1,850 (50.7%)	2,040 (47.7%)	2,130 (48.5%)	1,657 (46.2%)	1,454 (47.7%)	<0.001
Interventions														
PCI	648 (26.9%)	968 (34.1%)	1,012 (37.2%)	1,195 (40.2%)	1,366 (43.7%)	1,299 (44.9%)	1,559 (47.8%)	1,685 (49.6%)	1,935 (53.0%)	2,235 (52.2%)	2,995 (55.4%)	3,075 (56.1%)	3,270 (56.8%)	<0.001
CABG	186 (7.7%)	203 (7.2%)	202 (7.4%)	303 (10.2%)	240 (7.7%)	256 (8.8%)	191 (5.8%)	260 (7.6%)	225 (6.2%)	315 (7.4%)	250 (5.7%)	170 (4.8%)	185 (6.0%)	<0.001

CABG, coronary artery bypass graft; CAD, coronary artery disease; CKD, chronic kidney disease; PCI, Percutaneous coronary intervention; VF, ventricular tachycardia; VT, ventricular fibrillation.

**Table 2:** Trends in demographics, comorbidities, outcomes, and interventions in patients with STEMI and SCA, without diabetes.

Year	2005	2006	2007	2008	2009	2010	2011	2012	2013	2014	2015	2016	2017	*p*-trend
Age														
Mean Age (SD)	68 (14.0)	66 (14.0)	65 (15.0)	65 (15.0)	65 (14.0)	64 (14.0)	65 (14.0)	64 (14.0)	64 (14.0)	64 (14.0)	64 (13.0)	64 (14.0)	64 (13.0)	<0.001
Age: <55	1,910 (20.9%)	2,171 (24.2%)	2,132 (26.1%)	2,376 (26.3%)	2,066 (24.6%)	2,229 (27.2%)	2,115 (25.5%)	2,235 (26.8%)	2,025 (23.8%)	2,615 (27.1%)	2,320 (23.9%)	1,820 (24.8%)	1,920 (24.6%)	<0.001
Age: 55–64	1,922 (21.1%)	2,143 (23.9%)	1,928 (23.6%)	2,185 (24.2%)	2,248 (26.8%)	2,108 (25.8%)	2,233 (26.9%)	2,265 (27.1%)	2,420 (28.5%)	2,530 (26.2%)	2,770 (28.5%)	2,055 (28.0%)	2,315 (29.6%)	<0.001
Age: 65–74	1,911 (21.0%)	1,771 (19.7%)	1,556 (19.0%)	1,833 (20.3%)	1,729 (20.6%)	1,640 (20.0%)	1,597 (19.2%)	1,800 (21.6%)	1,890 (22.2%)	2,190 (22.7%)	2,285 (23.5%)	1,700 (23.1%)	1,950 (25.0%)	<0.001
Age: 75–84	2,274 (24.9%)	1,991 (22.2%)	1,702 (20.8%)	1,650 (18.3%)	1,428 (17.0%)	1,430 (17.5%)	1,502 (18.1%)	1,280 (15.3%)	1,395 (16.4%)	1,390 (14.4%)	1,510 (15.5%)	1,145 (15.6%)	995 (12.7%)	<0.001
Age: >85	1,101 (12.1%)	906 (10.1%)	862 (10.5%)	979 (10.9%)	923 (11.0%)	779 (9.5%)	859 (10.3%)	770 (9.2%)	765 (9.0%)	940 (9.7%)	830 (8.5%)	630 (8.6%)	635 (8.1%)	<0.001
Gender														
Male	5,677 (62.3%)	5,985 (66.6%)	5,337 (65.2%)	6,044 (67.0%)	5,590 (66.6%)	5,646 (69.0%)	5,633 (67.8%)	5,775 (69.2%)	5,860 (69.0%)	6,745 (69.8%)	6,775 (69.7%)	5,170 (70.3%)	5,720 (73.2%)	<0.001
Race														
Caucasians	5,450 (83.0%)	5,289 (82.7%)	4,675 (81.5%)	5,722 (81.7%)	5,842 (82.2%)	5,634 (80.4%)	5,737 (79.4%)	6,245 (80.2%)	6,290 (81.7%)	7,115 (79.2%)	7,315 (79.6%)	5,550 (80.6%)	5,665 (77.1%)	<0.001
African Americans	441 (6.7%)	432 (6.7%)	473 (8.2%)	452 (6.5%)	561 (7.9%)	612 (8.7%)	556 (7.7%)	580 (7.5%)	490 (6.4%)	845 (9.4%)	755 (8.2%)	685 (9.9%)	705 (9.6%)	<0.001
Hispanics	429 (6.5%)	362 (5.7%)	323 (5.6%)	365 (5.2%)	284 (4.0%)	345 (4.9%)	394 (5.5%)	470 (6.0%)	450 (5.8%)	530 (5.9%)	550 (6.0%)	295 (4.3%)	425 (5.8%)	<0.001
Asians	104 (1.6%)	144 (2.3%)	95 (1.6%)	159 (2.3%)	112 (1.6%)	193 (2.8%)	146 (2.0%)	170 (2.2%)	180 (2.3%)	205 (2.3%)	240 (2.6%)	155 (2.3%)	195 (2.7%)	<0.001
Comorbidities														
Hypertension	3,377 (37.0%)	3,353 (37.3%)	3,234 (39.5%)	4,146 (45.9%)	4,086 (48.7%)	3,903 (47.7%)	4,187 (50.4%)	4,240 (50.8%)	4,430 (52.1%)	5,420 (56.1%)	5,455 (56.2%)	4,505 (61.3%)	4,885 62.5%)	<0.001
Obesity	293 (3.2%)	315 (3.5%)	432 (5.3%)	515 (5.7%)	618 (7.4%)	536 (6.5%)	727 (8.8%)	845 (10.1%)	805 (9.5%)	1,100 (11.4%)	1,125 (11.6%)	860 (11.7%)	1,040 (13.3%)	<0.001
Smoking	1,712 (18.8%)	1,921 (21.4%)	1,961 (24.0%)	2,470 (27.4%)	2,853 (34.0%)	2,896 (35.4%)	3,083 (37.1%)	3,190 (38.2%)	3,365 (39.6%)	4,160 (43.0%)	3,785 (39.0%)	1,105 (15.0%)	1,235 (15.8%)	<0.001
Dyslipidemia	1,816 (19.9%)	1,900 (21.2%)	2,068 (25.3%)	2,635 (29.2%)	2,884 (34.4%)	2,782 (34.0%)	3,193 (38.4%)	3,405 (40.8%)	3,390 (39.9%)	4,025 (41.6%)	3,930 (40.5%)	3,155 (42.9%)	3,240 (41.5%)	<0.001
CAD	4,521 (49.6%)	4,805 (53.5%)	4,684 (57.3%)	5,880 (65.2%)	5,509 (65.6%)	5,511 (67.3%)	6,067 (73.0%)	6,260 (75.0%)	6,235 (73.4%)	7,310 (75.6%)	7,615 (75.9%)	8,230 (77.8%)	8,420 (80.5%)	<0.001
CKD	719 (7.9%)	923 (10.3%)	890 (10.9%)	763 (8.5%)	829 (9.9%)	729 (8.9%)	821 (9.9%)	795 (9.5%)	850 (10.0%)	970 (10.0%)	945 (9.7%)	830 (11.3%)	800 (10.2%)	<0.001
Secondary outcomes														
VT	2,265 (24.8%)	2,345 (26.1%)	1,989 (24.3%)	2,428 (26.9%)	2,074 (24.7%)	2,098 (25.6%)	2,258 (27.2%)	2,343 (26.8%)	2,225 (26.2%)	2,097 (27.1%)	3,670 (37.8%)	2,977 (40.5%)	3,204 (41.0%)	<0.001
VF	3,687 (40.4%)	3,799 (42.3%)	3,470 (42.4%)	4,031 (44.7%)	3,571 (42.5%)	3,938 (48.1%)	4,083 (49.1%)	4,145 (49.6%)	4,360 (51.3%)	4,900 (50.7%)	5,210 (53.6%)	4,075 (55.4%)	4,425 (56.6%)	<0.001
Acute renal failure	1,409 (15.5%)	1,506 (16.8%)	1,530 (18.7%)	1,742 (19.3%)	2,138 (25.5%)	2,144 (26.2%)	2,112 (25.4%)	2,290 (27.4%)	2,365 (27.8%)	2,940 (30.4%)	3,075 (31.7%)	2,390 (32.5%)	2,670 (34.2%)	<0.001
Cardiogenic shock	2,413 (26.5%)	2,567 (28.6%)	2,476 (30.3%)	2,979 (33.0%)	3,178 (37.9%)	3,094 (37.8%)	3,427 (41.3%)	3,425 (41.0%)	3,360 (39.6%)	4,240 (43.9%)	4,140 (42.6%)	4,285 (43.1%)	4,720 (47.1%)	<0.001
Interventions														
PCI	3,545 (38.9%)	4,057 (45.2%)	3,764 (46.0%)	4,635 (51.4%)	4,494 (53.5%)	4,556 (55.6%)	4,836 (58.2%)	5,235 (62.7%)	5,190 (61.1%)	6,095 (63.1%)	6,355 (65.1%)	7,020 (68.3%)	7,480 (69.7%)	<0.001
CABG	777 (8.5%)	704 (7.8%)	541 (6.6%)	646 (7.2%)	598 (7.1%)	545 (6.7%)	467 (5.6%)	510 (6.1%)	435 (5.1%)	445 (4.6%)	480 (4.9%)	280 (3.8%)	285 (3.6%)	<0.001

### 3.3. Comparison of both groups

Diabetes patients with SCA were older, more likely to be females, and belonged to ethnic minorities (Table 3, *p* < 0.001). They were also more likely to have comorbidities but smoked less. Diabetes was associated with a higher adjusted risk of mortality [aOR = 1.725 (1.621–1.837)], acute renal failure [aR = 1.529 (1.431–1.634)], and cardiogenic shock [aOR = 1.254 (1.179–1.333)] (Table 4). Unexpectedly, ventricular fibrillation was significantly lower in the diabetes group [aOR = 0.693 (0.652–0.737)]. Patients were also more likely to undergo revascularization with CABG [aOR = 1.197 (1.065–1.345)] but less likely to undergo PCI [aOR = 0.708 (0.664–0.754)].

**Table 3:** Comparison of demographics between SCA patients with diabetes vs. non-diabetes.

		Diabetes	Non-diabetes	*p* value
Age	Mean (SD)	66 (12.5)	64 (14.5)	<0.001
	<55	1,469 (17.1%)	5,635 (25.0%)	<0.001
	55–64	2,341 (27.2%)	5,887 (26.1%)	<0.001
	65–74	2,360 (27.4%)	4,823 (21.4%)	<0.001
	75–84	1,782 (20.7%)	3,981 (17.7%)	0.006
	>85	650 (7.6%)	2,206 (9.8%)	<0.001
Sex	Male	5,450 (63.4%)	15,339 (68.1%)	<0.001
	Female	3,152 (36.6%)	7,193 (31.9%)	<0.001
Race	Caucasians	5,257 (69.0%)	15,446 (80.6%)	<0.001
	African Americans	828 (10.9%)	1,535 (8.0%)	<0.001
	Hispanics	925 (12.1%)	1,060 (5.5%)	<0.001
	Asians	240 (3.1%)	429 (2.2%)	<0.001
Comorbidities	Hypertension	5,659 (65.8%)	10,159 (45.1%)	<0.001
	Obesity	1,639 (19.1%)	1,854 (8.2%)	<0.001
	Smoking	2,129 (24.8%)	6,805 (30.2%)	<0.001
	Dyslipidemia	4,065 (47.3%)	7,733 (34.3%)	<0.001
	CKD	2,094 (24.3%)	2,191 (9.7%)	<0.001
	CAD	5,109 (59.4%)	12,720 (57.5%)	0.039

**Table 4:** Cardiovascular events and the revascularization strategies in SCA patients.

Outcome	Number of Events (OR; 95% CI)		Adjusted OR (95% CI)	*p*-value
	No diabetes	Diabetes		
Mortality	9,538	4,767	1.725 (1.621–1.837)	<0.001
	(OR = 1)	(OR = 1.693; 1.611–1.78)		
Ventricular Tachycardia	7,440	2,733	0.997 (0.936–1.062)	0.923
	(OR = 1)	(OR = 0.945; 0.896–0.996)		
Ventricular Fibrillation	10,850	3,216	0.693 (0.652–0.737)	<0.001
	(OR = 1)	(OR = 0.643; 0.611–0.676)		
Acute Renal Failure	5,706	3,194	1.529 (1.431–1.634)	<0.001
	(OR = 1)	(OR = 1.742; 1.652–1.836)		
Cardiogenic Shock	8,024	3,526	1.254 (1.179–1.333)	<0.001
	(OR = 1)	(OR = 1.256; 1.194–1.322)		
PCI	11,048	3,465	0.708 (0.664–0.754)	<0.001
	(OR = 1)	(OR = 0.701; 0.667–0.737)		
CABG	1,358	606	1.197 (1.065–1.345)	0.003
	(OR = 1)	(OR = 1.182; 1.07–1.305)		

CABG, coronary artery bypass graft; PCI, percutaneous coronary intervention.

### 3.4. Predictors of mortality in patients with diabetes

The risk of mortality increases with age (Table 5). For instance, patients >85 years old have a 7-fold increased mortality risk [OR = 7.529 (6.578–8.011)], and so do females [OR = 1.694 (1.615–1.777)]. Compared to Caucasians, African-Americans, Hispanics, and Asians had higher mortality risk [ORs = 1.19 (1.093–1.296), 1.354 (1.235–1.485), 1.193 (1.023–1.392); respectively]. Comorbidities associated with increased mortality risk included hypertension, obesity, valvular heart disease, and renal failure. Paradoxically, smoking, dyslipidemia, and coronary artery disease had a protective effect.

**Table 5:** Predictors of mortality in STEMI patients with diabetes and SCA.

Parameters		OR (95% CI)
Age		
Age: <55		Reference
Age: 55–64		1.468 (1.371–1.572)
Age: 65–74		2.429 (2.266–2.603)
Age: 75–84		4.116 (3.823–4.433)
Age: >85		7.259 (6.578–8.011)
Gender		
Male		Reference
Female		1.694 (1.615–1.777)
Race		
White		Reference
Black		1.19 (1.093–1.296)
Hispanic		1.354 (1.235–1.485)
Asians		1.193 (1.023–1.392)
Comorbidities		
Hypertension	No	Reference
	Yes	1.076 (1.029–1.125)
Obesity	No	Reference
	Yes	1.693 (1.611–1.78)
Smoking	No	Reference
	Yes	0.728 (0.677–0.782)
Dyslipidemia	No	Reference
	Yes	0.552 (0.525–0.581)
Valvular heart Disease	No	ref
	Yes	1.434 (1.327–1.55)
Chronic kidney disease	No	Reference
	Yes	1.947 (1.824–2.08)
Coronary artery disease	No	Reference
	Yes	0.626 (0.598–0.655)

## 4. Discussion

The pathogenesis of SCA in patients with diabetes is believed to be due to acute atherosclerotic CAD. However, other mechanisms, including diabetic autonomic neuropathy, QT interval prolongation, diabetic cardiomyopathy, diabetic nephropathy, poor diabetes control, and hypoglycemia, may independently contribute to SCA. Diabetic autonomic neuropathy, a common complication of diabetes, is associated with a decrease in parasympathetic and an increase in sympathetic tone, increasing the susceptibility to arrhythmias. Patients with diabetic autonomic neuropathy may also develop electrocardiographic abnormalities such as QT prolongation, predisposing them to lethal ventricular arrhythmias (15, 16). Whitsel et al. showed that diabetes patients without diagnosed pre-existing heart disease in the upper quartile of the QT index (QTI > 107%) had a 3-fold increased risk of SCA (17). Diabetic autonomic neuropathy is also associated with resting tachycardia, exercise intolerance, intraoperative and perioperative cardiovascular instability, silent myocardial ischemia, and increased mortality (18). Diabetic cardiomyopathy is a condition in which pathologic changes in the myocardium develop without cardiovascular disease. It could contribute to ECG abnormalities, such as non-specific ST-T wave changes and QT prolongation. Autopsy of patients with diabetic cardiomyopathy revealed areas of myocardial fibrosis resulting from microvascular disease (19). Diabetic Nephropathy (DN) is the leading cause of end-stage renal disease and is associated with an increased risk of all-cause and cardiovascular mortality (20) The development of DN is characterized by activation of metabolic, inflammatory, and hemodynamic pathways by chronic hyperglycemia (21). The Rochester diabetic neuropathy study reported a 2-fold increase of sudden cardiac death (SCD) in patients with DN, independently of other comorbidities (22).

High HbA1c levels, associated with poor glycemic control, increase the risk of SCD (23, 24). Lastly, stringent glycemic control in diabetes patients often results in hypoglycemic episodes, which may also contribute to the pathogenesis of SCA. We have recently shown that hypoglycemia increases the risk of mortality and arrhythmias in equally both diabetes and non-diabetes STEMI patients (25). A meta-analysis of prospective studies by Goto et al. suggested increased cardiovascular risk with severe hypoglycemia (26). Hypoglycemia activates the sympathetic nervous response, producing catecholamine-mediated adverse effects on the myocardium. Catecholamine excess driven by hypoglycemia is also associated with platelet activation, leukocyte mobilization, and coagulation that may result in ischemic damage to the myocardium and endothelial dysfunction (27, 28).

Our study demonstrated that diabetes patients with STEMI were 1.7 times more likely to die from SCA than non-diabetes patients. This is consistent with previous studies which assessed diabetes and SCA in different populations other than STEMI. In the Paris Prospective Study I of middle-aged working-class men, the risk of SCD was increased in patients with diabetes (29). The prospective, population-based ARIC study in the US also found an association between diabetes and increased risk of SCD (30). Meta-analyses by Aune et al. and Zaccardi et al. suggested a 2-fold increase in the risk of SCD in those patients (31, 32). In a nationwide study in Denmark, Lynge et al. reported a significantly higher incidence rate of SCD in diabetic patients within 1–49 years of age than in non-diabetic patients of the same age (33).

Risk factors associated with increased mortality also align with previous studies that assessed the link between diabetes and SCA. The mortality risk increased with age, consistent with previous studies (3, 34, 35). Although male patients predominantly experienced SCA in the setting of diabetes, the mortality risk was higher among female patients. In the Framingham study, the association between SCA and diabetes was more robust in females (36). In a case-control study by Escobedo et al., diabetes was associated with SCD in individuals with pre-existing coronary heart disease, and women with a history of diabetes were more strongly associated with SCD than men (37).

It has already been shown that mortality rates are different among ethnicities. Benjamins et al. reported that all-cause mortality is 20% higher in African-Americans compared to Caucasians (38). The increased mortality due to SCA among African-American patients has also been described previously (3, 34, 35, 39). Increased odds of cardiovascular mortality have also been reported in South East Asians (40). The higher mortality rates among non-Caucasians may be attributed to socioeconomic and predisposing factors (41). The increased prevalence of comorbidities among non-Caucasians may predispose those patients to poorer outcomes (42–44). Pharmacogenetics and personalized medicine based on genetic clusters might help reduce CVD risk disparities among different ethnicities (45). Race and genetic information are progressively shown to be important determinants of risk prediction (46). Further, current CVD guidelines emphasize the importance of ethnic differences in treating cardiovascular risk factors. The HELIUS study recently reported a beneficial effect of earlier hypertension treatment in ethnic minorities compared to Caucasians (47).

Interestingly, cardiovascular risk factors, including smoking, dyslipidemia, and CAD, had a protective effect, a finding that we and others already reported in the NIS database (9, 10, 12, 25, 48, 49). The paradoxical protective effect may be due to confounding factors not accounted for in our study, such as medications. Patients with known CAD may be more likely to undergo regular screening and follow-up for diabetes control and cardiovascular disease. However, it has been shown that hibernating myocardium in chronic CAD induces sympathetic remodeling and nerve sprouting, which were associated with VF and VT (50, 51). Further, myocyte adaptation in the hibernating myocardium, secondary to chronic ischemia, results in a substrate with enhanced vulnerability to lethal arrhythmias and SCD (52).

The decline in mortality related to SCA could be due to advances in cardiopulmonary resuscitation and post-resuscitation care (53), and potentially due to novel hypoglycemic agents such as SGLT-2 inhibitors and GLP-1 agonists. Those medications have been implicated in reducing the risk of cardiac arrhythmias. SGLT-2 inhibitors have a pleiotropic effect in patients with diabetes, irrespective of their impact on glycemic control (54). In a recent meta-analysis of over 30 studies, SGLT-2 inhibitors reduced the risk of arrhythmias by almost 20% (55). Although the exact mechanisms are not clear, it is believed that SGLT-2 inhibitors improve the electrical stability of the heart by decreasing sympathetic activity and attenuating the pro-arrhythmogenic sodium current (late I_Na_) (56). However, there is conflicting data on the effectiveness of these agents in the risk reduction of SCA and SCD in particular. A meta-analysis of 34 randomized controlled trials by Fernandes et al. suggested that treatment with an SGLT2 inhibitor was associated with a decreased risk in patients with diabetes (55). Whereas other meta-analyses by Li et al. and Sfairopoulos et al. showed a reduction in the risk of SCD in diabetic patients with SGLT-2 inhibitors, this reduction was not statistically significant (57, 58).

Similarly, a meta-analysis by Liu et al. of 5 randomized controlled trials suggested that there was no statistically significant difference in the risk of SCD between diabetes patients treated with GLP-1 receptor agonists and the placebo group (59). Lipid-lowering agents and statins are commonly used in patients with dyslipidemia, peripheral vascular disease, and CAD. Statins have been implicated in having antiarrhythmic properties in several studies (60). Statin use could underlie the protective effect of these comorbidities.

The results of our study must be interpreted within its limitations. The data analyzed in our study was extracted using ICD-9-CM and ICD-10-CM codes; hence, the transition could have led to extraction errors and variations in the number of cases and events. Additionally, the results are subject to administrative errors, such as issues with reporting and coding inaccuracies. As the NIS data relies on discharge diagnoses, the data lacks in-hospital temporality. Furthermore, due to the unavailability of patient-level data, factors such as medications, glycemic control, and duration and severity of comorbidities could not be included.

Further, the NIS is a retrospective observational database, and due to the absence of randomization, only correlations can be made, and no causality can be established. Our analysis only included hospitalized patients, which may represent a sicker cohort with an increased prevalence of adverse outcomes compared to the general population. Lastly, due to the unavailability of data following patient discharge or readmission data, follow-up of patients was not possible. Despite these limitations, our results are derived from a large sample representative of the US population, which is ideal for assessing temporal trends and potential associations.

## 5. Conclusion

Although diabetes is associated with an increased risk of sudden cardiac arrest in ST-elevation myocardial infarction, in-hospital mortality in those patients is declining despite an increase in cardiometabolic comorbidities. While decreasing mortality is reassuring, more has to be done since diabetes is still associated with worse cardiovascular outcomes.

## Data Availability

The raw data supporting the conclusions of this article will be made available by the authors, without undue reservation.
